# Genome-Wide Methylation Mapping Using Nanopore Sequencing Technology Identifies Novel Tumor Suppressor Genes in Hepatocellular Carcinoma

**DOI:** 10.3390/ijms22083937

**Published:** 2021-04-11

**Authors:** Colin F. Davenport, Tobias Scheithauer, Alessia Dunst, Frauke Sophie Bahr, Marie Dorda, Lutz Wiehlmann, Doan Duy Hai Tran

**Affiliations:** 1 Research Core Unit Genomics OE 9415, Medizinische Hochschule Hannover, Carl-Neuberg-Str. 1, D-30623 Hannover, Germany; Davenport.Colin@mh-hannover.de (C.F.D.); tobias.scheithauer@icloud.com (T.S.); Boehm.Marie@mh-hannover.de (M.D.); Wiehlmann.Lutz@mh-hannover.de (L.W.); 2 Institut fuer Zellbiochemie, OE4310, Medizinische Hochschule Hannover, Carl-Neuberg-Str. 1, D-30623 Hannover, Germany; alessi2012@gmail.com (A.D.); Bahr.Frauke@mh-hannover.de (F.S.B.)

**Keywords:** nanopore sequencing, methylation, tumor suppressor gene, glucose metabolism, hepatocellular carcinoma

## Abstract

Downregulation of multiple tumor suppressor genes (TSGs) plays an important role in cancer formation. Recent evidence has accumulated that cancer progression involves genome-wide alteration of epigenetic modifications, which may cause downregulation of the tumor suppressor gene. Using hepatocellular carcinoma (HCC) as a system, we mapped 5-methylcytosine signal at a genome-wide scale using nanopore sequencing technology to identify novel TSGs. Integration of methylation data with gene transcription profile of regenerated liver and primary HCCs allowed us to identify 10 potential tumor suppressor gene candidates. Subsequent validation led us to focus on functionally characterizing one candidate—glucokinase (GCK). We show here that overexpression of GCK inhibits the proliferation of HCC cells via induction of intracellular lactate accumulation and subsequently causes energy crisis due to NAD+ depletion. This suggests GCK functions as a tumor suppressor gene and may be involved in HCC development. In conclusion, these data provide valuable clues for further investigations of the process of tumorigenesis in human cancer.

## 1. Introduction

Hepatocellular carcinoma (HCC) is one of the most prevalent tumor types worldwide [1]; however, current treatment options are limited, and there are no precise and effective medical strategies [2]. HCC typically occurs on a background of chronic liver disease, with risk factors including viral or autoimmune hepatitis, chronic alcohol abuse, and nonalcoholic fatty liver disease [3,4]. These risk factors trigger aberrant liver regeneration, which initiates the formation of HCC. However, the underlying molecular mechanism is still largely unknown. Recent exome sequencing on normal, precancerous, and progressive HCC identified 161 putative driver mutations [5]. Importantly, none were detected in precancerous tissue, suggesting other alterations are required to drive HCC formation [5]. Dysregulated DNA methylation is reported to be one of the early events in HCC pathogenesis [6]. Furthermore, recent data from us and others show that suppression of multiple TSGs such as CDKN2A, HNF4A, or TTP via promoter hypermethylation plays a key role in the proliferation of HCC cells and regenerated hepatocyte [7,8,9,10], suggesting dysregulation of TSG expression via promoter methylation might be an important factor in HCC formation. Thus, the identification of novel TSGs will provide clues to delineate the molecular mechanism of HCC formation.

By far, 5-methylcytosine (5mC) and 5-hydroxymethylcytosine (5hmC) are the two most common epigenetic marks found in the mammalian genome. Moreover, 5mC suppresses gene transcription when present at elevated levels in gene promoters [11,12], and 5hmC is generated from 5mC by the ten-eleven translocation (TET) family of dioxygenases [13]. Recent data suggested 5hmC may be an intermediate of 5mC oxidation product and is also steadily incorporated into the genomic DNA with high stability [14]. Notably, replacing 5mC with 5hmC in the promoter region can reactivate gene transcription [15]. Thus, it is crucial to distinguish between methylated and hydroxymethylated promoters. The current “gold standard” method for profiling methylation in DNA is bisulfite sequencing, which yields the sum of methylation (5mC) and hydroxymethylation (5hmC) [16]. The recently developed nanopore sequencing technology allows the detection of 5mC signals directly from native DNA strands [17,18]. It records sequence-dependent changes in ionic current over time as single-stranded DNA passes through a protein nanopore inserted in a lipid membrane [19]. 

In this study, we applied Oxford nanopore sequencing technology to map the 5-methylcytosine signal across the HCC genome. Further integration of methylation data with transcriptome profile of regenerated liver and primary HCCs generated by the Cancer Genome Atlas (TCGA) allowed identification of potential hypermethylated tumor suppressor gene candidates. Finally, we functionally characterized one candidate—glucokinase (GCK)—as a tumor suppressor gene in HCC cells. 

## 2. Results 

### 2.1. Identification of Hypermethylated Genes in HCC Cells

**Figure 1:**
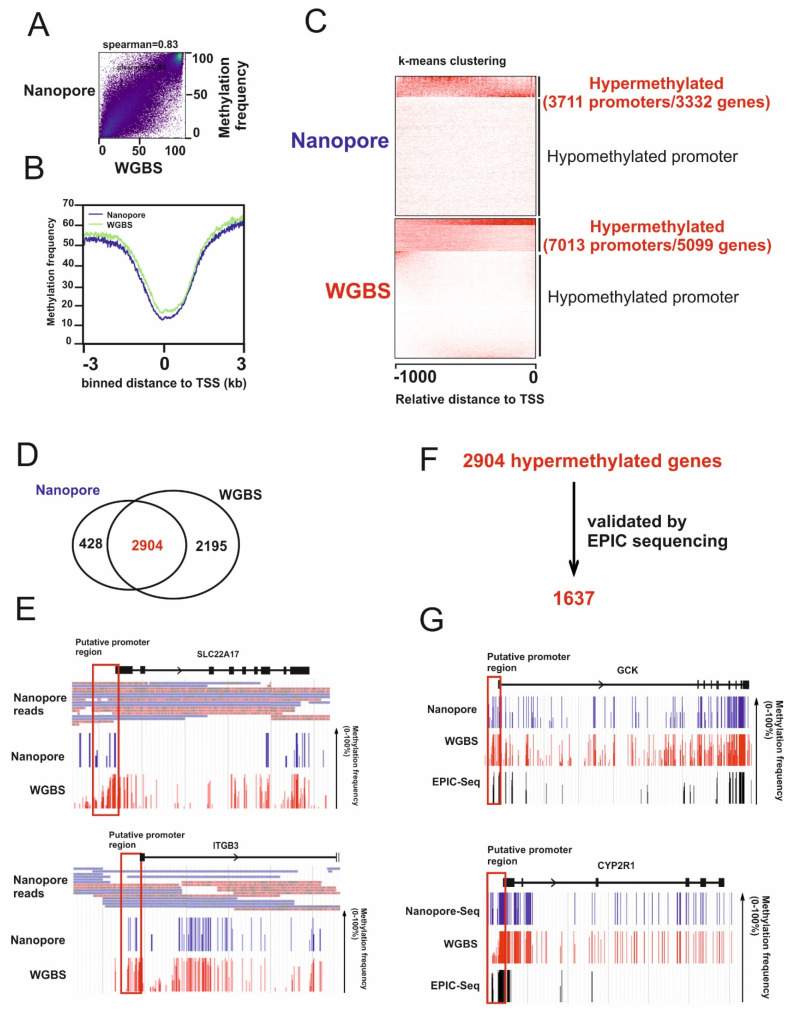
Identification of hyper-5mC genes in hepatocellular carcinoma (HCC). (**A**) Scatterplot showing the correlation between methylation frequencies generated by nanopore sequencing versus WGBS. (**B**) Metaplot of the nanopore and whole-genome bisulfite sequencing (WGBS) methylation calls around annotated transcription start sites (TSSs). (**C**) k-means clustering on 29598 promoters of 16455 genes (Eukaryotic Promoter Database) was performed for methylation calls generated by nanopore and WGBS. (**D**) Common hypermethylated genes detected by nanopore and WGBS. (**E**) Nanopore read coverage and methylation profile of glucokinase (GCK) and CYP2R1 gene generated by nanopore and WGBS were visualized using JBrowse. (**F**) A total of 1637 hypermethylated genes detected by nanopore and WGBS were confirmed by EPIC sequencing. (**G**) Methylation profile of GCK and CYP2R1 gene generated by nanopore, WGBS, and EPIC sequencing was visualized using JBrowse. Red boxes indicate putative promoter regions.

To map the 5mC signal at a genome-wide scale in HnCC cells, we sequenced an HCC cell line (HepG2) on the MinION platform to a depth of ~3x and called a 5mC signal using *nanopolish* [17]. First, we compared methylation signal generated by nanopore sequencing with whole-genome bisulfite sequencing (WGBS) of HepG2 cells generated by the ENCODE Consortium (SRA accession SRR4235786). As shown in Figure 1A, the methylation signal generated by nanopore sequencing and WGBS was well correlated (Spearman correlation = 0.83). The genome-wide methylation data generated by nanopore sequencing successfully recapitulated known patterns around promoter regions. For example, the methylation level dipped at transcriptional start sites (TSSs) (Figure 1B). To identify TSG candidates, we focused on 29598 human promoters [20]. CpG sites that have a methylation frequency of more than 50% were selected for promoter analysis. We clustered hypermethylated CpG sites into hyper- and hypomethylated promoters using the Eukaryotic Promoter Database at http://epd.vital-it.ch (accessed on 30 April 2019) [21]. A total of 3711 and 7013 hypermethylated promoters of 3332 and 5099 genes were detected using nanopore sequencing and WGBS (Figure 1C). In agreement with correlation analysis (Figure 1A,B), 87% of hypermethylated genes (2904 genes) detected by nanopore sequencing were also detected by WGBS (Figure 1D). Since WGBS yields the sum of methylation (5mC) and hydroxymethylation (5hmC), we analyzed nanopore read coverage of 2195 hypermethylated genes detected only by WGBS to identify possible hydroxymethylated genes. Promoters of 489 out of 2195 genes have at least five nanopore reads, yet no 5mC- or low level of 5mC methylation was detected by *nanopolish*, suggesting they might be hydroxymethylated (Figure 1E, Table S1). These data also indicate 5mC calling from nanopore reads is able to distinguish 5mC from other modifications of cytosine. We also note that some of the 428 genes in which methylation was detected solely by nanopore were in regions of the genome inaccessible to Illumina short-read sequencing. On the other hand, the greater coverage of Illumina sequencing undoubtedly increased its power to detect methylation across the genome.

To extract highly confident methylation signals further, we performed deep bisulfite sequencing, targeting 3.3 of approximately 30 million CpG sites (EPIC sequencing, Illumina) for our HepG2 cells. Out of 2904 hypermethylated genes detected by both nanopore and WGBS, 1637 could be confirmed by EPIC sequencing (Figure 1F,G, Table S2).

### 2.2. Identification of Candidates for Novel TSGs in HCC

**Figure 2:**
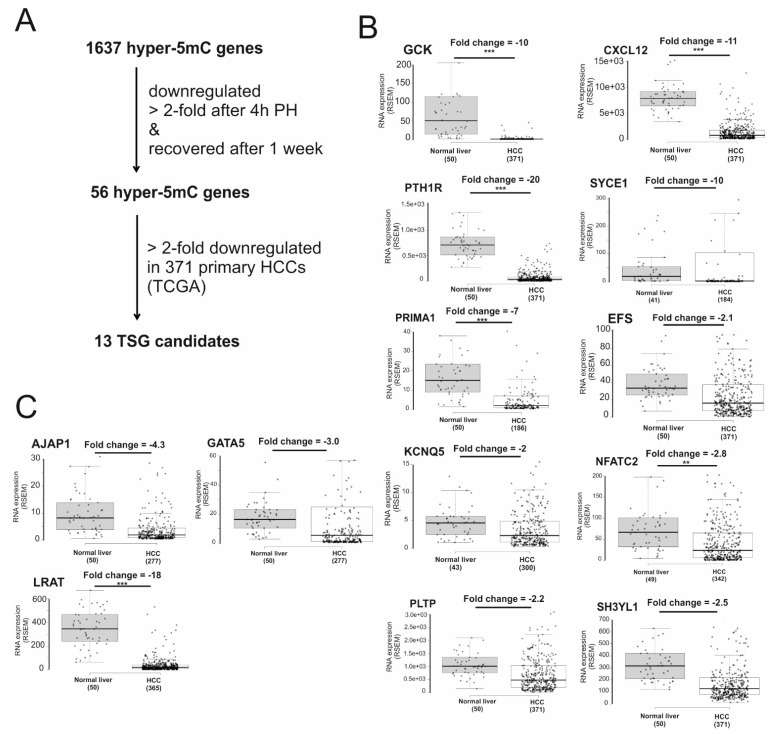
Identification of tumor suppressor gene (TSG) candidates. (**A**) Strategy to identify potential TSGs: gene expression of 1637 hypermethylated genes before and four hours (4 h) after partial hepatectomy (PH) was examined using previously published RNA-Seq data (GSE95135). To narrow TSG candidates further, the expression of 56 genes downregulated after PH in 371 primary HCCs was examined. (**B**,**C**) Boxplot showing gene expression of 13 TSG candidates in normal liver versus primary HCC: mRNA expression generated by the Cancer Genome Atlas (TCGA) and fold change (normal liver versus HCC) were retrieved from Firebrowse (http://firebrowse.org/, accessed on 3 February 2021) data portal. **: *p*-value < 0.0001; *** *p*-value < 0.0000001. RSEM: RNA-Seq by expectation–maximization [35].

During liver regeneration, proproliferative genes are activated and antiproliferative genes are suppressed to allow quiescent hepatocytes to re-enter the cell cycle. Since aberrant liver regeneration initiates HCC formation [22,23,24], we examined the expression level of hypermethylated genes before and four hours after two-thirds partial hepatectomy (PH) in mouse to narrow down TSG candidates [25]. We focused on genes that were downregulated more than twofold, compared to control sham surgery (SH), with the added criterion that expression of these genes should have recovered one week post partial hepatectomy (Figure 2A). Based on these criteria, 56 genes were selected (Table S3). To narrow down candidates for tumor suppressor genes further, we compared the expression of these genes in 371 human primary HCCs and 50 normal liver using RNA-Seq data generated by the TCGA project [26]. Out of 56 genes, 13 were downregulated by more than twofold in HCC, compared to normal liver (Figure 2B,C). Of these 13 gene candidates, three genes are already known to be tumor suppressor genes (Figure 2C)—adherens junctions associated protein 1 (AJAP1) [27,28], GATA binding protein 5 (GATA5) [29,30,31,32], and lecithin retinol acyltransferase (LRAT) [33,34]. These TSGs are known to be silenced by DNA methylation in HCC, indicating the success of our methodology. Furthermore, 10 potential tumor suppressor genes have diverse functions (Table 1), including transcriptional regulation (NFATC2), cell migration (EFS and CXCL12), glucose metabolism (GCK), and cell growth (PTH1R and SH3YL1).

### 2.3. Ectopic Expression of GCK Inhibits Proliferation of HCC Cells 

**Figure 3:**
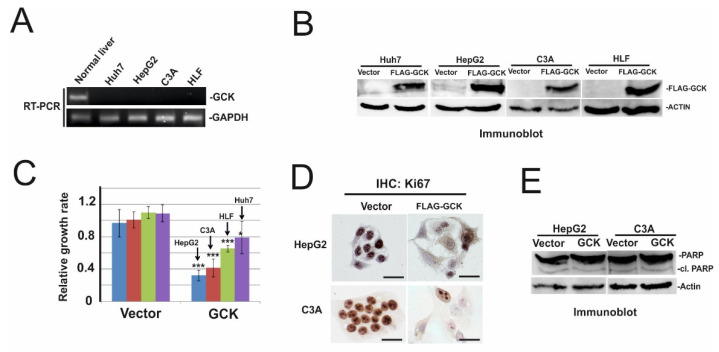
Overexpression of GCK inhibited cell proliferation. (**A**) Total RNAs from normal human liver (Origene), Huh7, HepG2, C3A, and HLF cells were used to examine the expression of GCK. glyceraldehyde-3 phosphate dehydrogenase (GAPDH) was used as the loading control. (**B**) Control vector (Vector) and FLAG-tagged GCK (FLAG–GCK) were overexpressed in Huh7, HepG2, C3A, and HLF cells. Two days after transfection, total cell lysates were applied for FLAG and actin-specific immunoblot. Actin was used as the loading control. (**C**) Sister cultures of (B) were subjected to crystal violet assay. Numbers are mean ± SD. ***: *p*-value < 0.001, *: *p*-value < 0.05. (**D**) HepG2 and C3A cells were transfected with the control vector and FLAG–GCK. Two days after transfection, cells were fixed in ice-cold methanol and stained with Ki67 specific antibody by IHC (immunohistochemistry) technique. Representative images were shown. Bars represent 50 µm. (**E**) Sister culture of (**D**) was subjected to PARP and actin-specific immunoblotting. Actin was used as the loading control.

Since reprogramming of glucose metabolism is one of the hallmarks of HCC [46], we further focus on characterizing the antiproliferative effect of one TSG candidate—glucokinase (GCK). Glucokinase is an isoenzyme of the mammalian hexokinase group. In the liver, glucokinase plays a key regulatory role in glucose uptake. Due to its high Km and specificity for glucose and the lack of product inhibition by glucose-6-phosphate, glucokinase ensures glucose utilization by hepatocytes [38]. To examine whether GCK may function as a tumor suppressor gene in HCC cells, we first analyzed the expression of GCK in normal human liver and four HCC cell lines, namely, Huh7, HepG2, C3A cells, and HLF cells (Figure 3A). In agreement with TCGA data (Figure 2B), GCK is highly expressed in normal liver and not detected in Huh7, HepG2, C3A cells, and HLF cells. We then overexpressed FLAG-tagged GCK in these cell lines (Figure 3B) for two days and examined cell growth using a crystal violet assay. Overexpression of GCK strongly inhibited the growth of HepG2 and C3A cells (>twofold inhibition), while the effect of growth inhibition was less pronounced but still significant in HLF and Huh7 cells (1.5- and 1.3-fold, respectively) (Figure 3C). Growth inhibition upon GCK overexpression in HepG2 and C3A cells was confirmed by immunohistochemical staining of Ki67, a proliferation marker (Figure 3D).

To examine whether the reduction of HepG2 and C3A cells upon GCK overexpression is due to inhibition of proliferation and/or apoptotic induction, we performed immunoblotting of PARP (Poly (ADP-Ribose)-Polymerase), an apoptotic marker. As shown in Figure 3E, the cleavage of PARP was not increased in GCK overexpressing cells. These data suggest overexpression of GCK inhibits cell proliferation but does not induce apoptosis.

### 2.4. Ectopic Expression of Glucokinase Caused Intracellular Lactate Accumulation and Induced Energy Crisis Due to NAD+ Depletion

**Figure 4:**
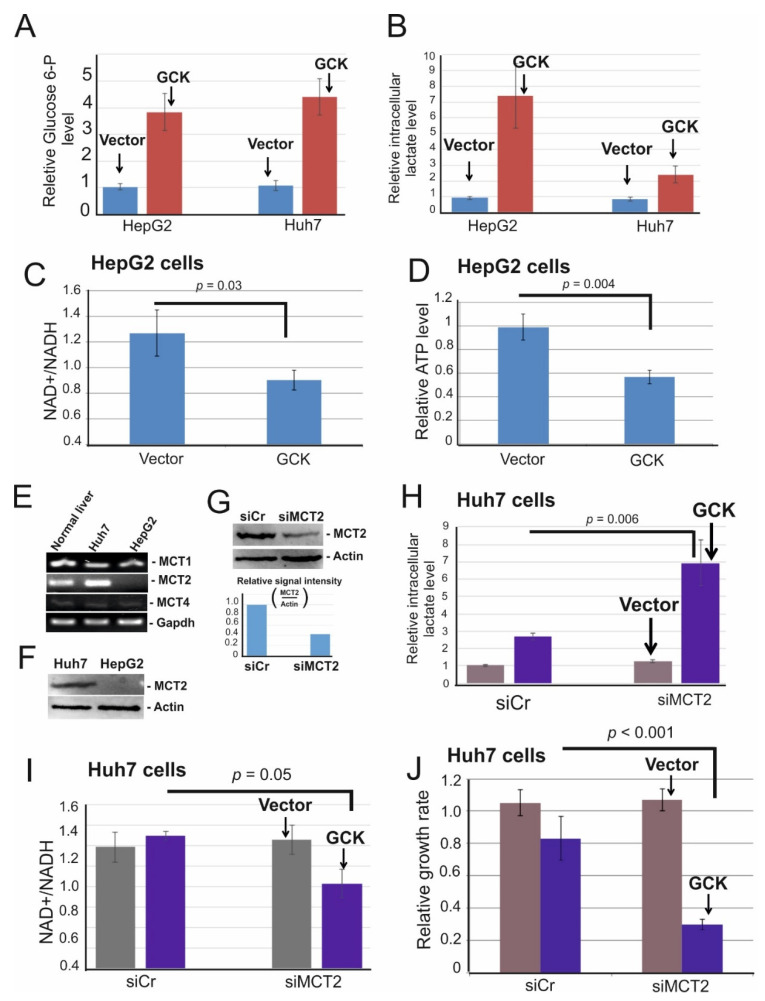
GCK overexpression caused intracellular lactate accumulation and induced energy crisis due to NAD+ depletion. (**A**,**B**) Huh7 and HepG2 cells were transfected with a control vector (Vector) and FLAG-tagged GCK. Two days after transfection cells were homogenized and deproteinized. Deproteinized lysates were then supplied for glucose-6-phosphate assay (**A**) or lactate assay (**B**). (**C**,**D**) HepG2 cells were transfected with a control vector (Vector) and FLAG-tagged GCK. Huh7 and HepG2 cells were used to examine the expression of MCT1-4. GAPDH was used as the loading control. (**F**) Cell lysates of Huh7 and HepG2 cells were applied for MCT2 specific immunoblot. Actin was used as the loading control. (**G**) Huh7 cells were transfected with siRNA control (siCr) and siMCT2. Three days after transfection cells were lysed and subjected to MCT2 and actin-specific immunoblotting. Immunoblot signal was quantified using ImageQuant. (**H**,**I**) Huh7 cells were transfected with control siRNA (siCr) and siMCT2. After 24 hours, cells were transfected with vector control and FLAG–GCK and further cultured for two days. Cells were then supplied for lactate- (**H**) and NAD+/NADH assay (**I**) as described above. (**J**) Sister cultures of (J) were subjected to crystal violet assay. Numbers are mean ± SD. *p*: *p*-value.

It has been shown that overexpression of glucokinase in rat hepatoma cells cultured in high-glucose media-induced glucose uptake and high levels of lactate accumulated [47]. Notably, our cells were also cultured in high glucose (25 mM) media. Thus, we examined levels of intracellular glucose-6-phosphate (Glucose 6-P) and lactate in control- and GCK overexpressing cells. Although the level of Glucose 6-P increased similarly upon GCK overexpression in both HepG2 and Huh7 cells (Figure 4A), the intracellular lactate is markedly accumulated only in HepG2 (7.4 fold) but not Huh7 cells (2.4 fold) (Figure 4B). The conversion of pyruvate to lactate-by-lactate dehydrogenase plays a key role in the regeneration of NAD+ [48]. Notably, at a high concentration of lactate, the lactate dehydrogenase exhibits feedback inhibition, and the rate of NAD+ regeneration is decreased [47]. NAD+ is reduced to NADH during glycolysis, and a constant supply of NAD+ is required to maintain a continuously high rate of glycolysis and optimal cell growth [49]. A drop in the NAD+/NADH ratio correlates with lower cell proliferation [49]. Thus, we examined the influences of intracellular lactate accumulation on the regeneration of NAD+ and ATP production. As shown in Figure 3C, the NAD+/NADH ratio dropped significantly in GCK overexpressing cells and was concomitant with reduced ATP levels (Figure 4D), suggesting intracellular accumulation of lactate-induced energy crisis due to NAD+ depletion. 

### 2.5. MCT2 Is Required to Reduce Intracellular Lactate Accumulation in GCK Overexpressing Cells

Since GCK overexpression did not strongly induce lactate accumulation in Huh7 cells, it raises the question of how lactate is effluxed in these cells. The efflux of lactate is mediated by the bidirectional monocarboxylate transporters (MCTs) [50]. Four MCTs are known to transport lactate. Thus, we examined the expression of MCT variants in Huh7 and HepG2 cells. MCT3 is not expressed in both cell lines (data not shown). Interestingly, HepG2 cells express only MCT1 and MCT4, while Huh7 cells express all three variants (Figure 4E). We also confirmed the lack of MCT2 in HepG2 cells by immunoblotting (Figure 4F). These data raise the subsequent question of whether intracellular lactate accumulation is due to the lack of MCT2 in HepG2 cells. To examine this possibility, we depleted MCT2 in GCK overexpressing Huh7 cells (Figure 4G) and measured the concentration of intracellular lactate. Indeed, the depletion of MCT2 markedly induced lactate accumulation by sevenfold (Figure 4H). Consequently, the NAD+/NADH ratio significantly dropped in MCT2 depleted cells (Figure 4I) and concomitant with reduced cell proliferation (Figure 4J). In summary, these data indicate that the lack of MCT2 strongly accumulated intracellular lactate in GCK overexpressing cells. 

## 3. Discussion

Loss-of-function mutation of TSG or downregulation of expression via promoter hypermethylation or gene depletion plays a key role in cancer formation. Recent evidence has accumulated that TSGs also function as metabolic regulators to modify cellular metabolism to support enhanced cell growth and proliferation or engage strategies of metabolic adaptation to survive periods of metabolic stress [51,52].

Aberrant liver regeneration caused by viral or autoimmune hepatitis, chronic alcohol abuse, and nonalcoholic fatty liver initiates HCC formation. However, the underlying mechanism remains largely unknown. It has been recently shown that suppression of tumor suppressor genes via promoter hypermethylation might play a key role in hepatocarcinogenesis [6,9,10]. Although several genome-wide methylation studies applying methylation array were conducted to identify tumor suppressor genes in HCC, this technology is based on bisulfite conversion, which fails to discriminate between methylation and hydroxymethylation [33,53,54]. Notably, replacing hypermethylated promoters with hydroxymethylated promoters has been shown to activate gene transcription [15,55]. Thus, alternative methods are required to distinguish between methylation and hydroxymethylation.

In this study, we applied nanopore sequencing technology to identify novel hypermethylated TSG in HCC. This novel technology allows us to map methylation signals at a genome-wide scale. Our data show that the methylation signal generated by nanopore sequencing correlated well with WGBS (Figure 1AB). We also show that this technology is able to distinguish 5mC from other modifications of cytosine such as 5hmC (hydroxymethylation) (Figure 1E, Table S1). This suggests nanopore sequencing is a suitable method to identify hypermethylated TSGs. 

To narrow down candidates for hypermethylated TSG, we integrated our methylation data with gene expression profiles from regenerated liver and primary HCCs (TCGA). Since transcriptome profiling data of human liver regeneration is not available at present, we analyzed gene expression profiling of hypermethylated genes during mouse liver regeneration using previously published RNA-Seq data [25], despite the fact that approximately one percent of human genes do not have a mouse orthologue and vice versa [56]. To our knowledge, we are the first to use this particular combined approach to identify tumor suppressor genes that can be functionally validated. With this approach, we identified 13 candidate tumor suppressor genes, including AJAP1, GATA5, and LRAT, which were previously reported to be TSG and silenced by DNA methylation in HCC [29,33,57]. Moreover, 10 novel TSG candidates have diverse molecular functions (Table 1), such as embryonal Fyn-associated substrate (EFS) involved in integrin-dependent signaling pathways [37], proline-rich membrane anchor 1 (PRIMA1) regulating the assembly of acetylcholinesterase [58], or glucose metabolism (GCK) [38]. Since integrin β_3_ is a TSG in HCC [59,60], the role of EFS in integrin β_3_ dependent-signaling remains to be studied. It has been shown that the degradation of acetylcholine by acetylcholinesterase directly promotes growth inhibition by inactivating mitogen-activated protein kinase and phosphatidylinositol-3′-phosphate kinase/protein kinase B pathways in HCC cells [61]. This suggests PRIMA1 may function as a TSG via the regulation of acetylcholinesterase. 

Since glucose metabolism reprogramming is one of the hallmarks of HCC, we functionally characterized one candidate—glucokinase (GCK)—as a tumor suppressor gene. Hexokinases catalyze the first committed step in glucose metabolism by phosphorylating glucose. In normal differentiated hepatocytes, GCK is the major hexokinase (HK) isoform expressed, while in HCC, GCK expression is repressed and expression of the fetal HK isoform, HK2, is induced [62]. Compared to HK2, GCK has a low affinity to glucose and is not inhibited by its product, glucose-6-phosphate, which is adapting hepatocyte function to glycemia [38]. Conversely, HCC cells express the high-affinity HK2 to sustain tumor proliferation even at low glucose concentrations. The molecular mechanism by which GCK is suppressed in HCC and its consequences are still largely unknown. 

We show here that the expression of GCK is suppressed due to promoter hypermethylation (Figure 1G). Furthermore, GCK antagonizes the growth of HCC cells (Figure 3). Since overexpression of GCK does not influence enzyme activity of HK2 [47], downregulation of GCK per se may participate in HCC formation. Overexpression of GCK induces a high level of glucose 6-P. Due to the high level of glucose 6-P, aerobic glycolysis is increased [63], which leads to intracellular lactate accumulation in HCC cells and thereby impairs NAD+/NADH regeneration in cells lacking monocarboxylate transporter 2 (MCT2) (Figure 4BC). Consequently, impaired NAD+/NADH regeneration leads to reduced ATP production and induces growth arrest. The bidirectional monocarboxylate transporters (MCTs) prevent intracellular acidification by mediating the efflux of L-lactate in high glycolytic cells [50]. Notably, among MCTs, MCT2 has the highest affinity to L-lactate [50], suggesting MCT2 may be required to reduce lactate accumulation in GCK overexpressing cells. Indeed, we show that GCK induced intracellular lactate accumulation is much less pronounced in HCC cells expressing MCT2 (Figure 4E-J). Notably, recent immunohistochemical analysis of MCT1, MCT2, and MCT4 on 73 primary HCCs revealed that the plasma membrane expression of MCT2 was detected in only 12.2% of tumor samples, while MCT1 and MCT4 were detected in 54.8% and 38.2% of tumor samples, respectively [64]. These data suggest that the expression of GCK might be suppressed during hepatocarcinogenesis due to the loss of MCT2.

Does GCK function as a TSG in other types of cancer? GCK activity was detected in several tumor cell lines derived from colon cancer (HT29 cells), melanoma (RVH 421 cells), or urinary bladder carcinoma (RT112 cells). The authors show that the treatment of D-Mannoheptulose inhibited the growth of these tumor cells [65]. D-Mannoheptulose is a hexokinase inhibitor. It has been recently shown that it inhibits GCK and other isoforms of hexokinase such as HK2 [66,67]. Thus, the role of GCK in other types of cancer remains to be studied.

In summary, by applying nanopore sequencing technology we profiled a highly confident 5-methylcytosine signal across the HCC genome and identified biologically meaningful tumor suppressor gene candidates, which are epigenetically silenced in HCC. This work has led to the discovery and characterization of one gene in HCC that behaves as a tumor suppressor gene and provided multiple candidates for future analysis.

## 4. Materials and Methods

### 4.1. Cell Culture, siRNA, and Transfection

HepG2, Huh7, C3A, and HLF cells were purchased from the American Type Culture Collection (ATCC) (Manassas, VA, USA) or the DMSZ-German collection of microorganisms and cell culture (Braunschweig, Germany). They were grown in high glucose DMEM (Dulbecco’s Modified Eagle Medium) supplemented with 10% FCS (fetal calf serum). All cell lines are free of mycoplasma contamination.

Control *siRNA* (5′-UAAGGCUAUGAAGAGAUAC-3′), *siMCT2* (5′-AUGAUAAUUAUUACAUAUAGA-3′) were from Microsynth AG (Balgach, Switzerland). A total of 50 pmol of each *siRNA* were transfected using TransfeX™ (ATCC HB-8065TM, ATCC). For ectopic expression of GCK experiments, the GCK coding sequence was cloned into pcDNA3.1+/C-(K)-DYK plasmid and purchased by Genscript (Piscataway, NJ, USA). Plasmids were transfected using TransfeX™.

### 4.2. Immunohistochemistry (IHC)

Immunohistochemical studies were performed as detailed previously [68]. Rabbit monoclonal anti Ki67 was purchased from Thermo Scientific (Waltham, MA, USA).

### 4.3. Immunoblotting Procedures

Details of immunoblotting have been described previously [10]. Polyclonal antibody against actin was purchased from Santa Cruz Biotechnology. Polyclonal rabbit anti-FLAG and PARP antibodies are from Proteintech (Rosemont, IL, USA) and Cell Signaling Technology (Cambridge, UK), respectively. Mouse monoclonal anti MCT2 (SLC16A7) was purchased from Santa Cruz Biotechnology (Dallas, TX, USA). Corresponding proteins were visualized by incubation with peroxidase-conjugated anti-rabbit, anti-mouse, or anti-goat immunoglobulin (Santa Cruz Biotechnology), followed by incubation with SuperSignal West FemtoMaximum Sensitivity Substrate (Pierce (Rockford, IL, USA)). Results were documented on a LAS4000 imaging system (GE Healthcare Bio-Sciences (Uppsala, Sweden)).

### 4.4. DNA and RNA Extraction

RNA was isolated from cells with the High Pure RNA Isolation kit (Roche Diagnostics (Rotkreuz, Switzerland)) according to the manufacturer´s instructions. Genomic DNA (gDNA) was isolated using QIAamp DNA Kits (Qiagen (Hilden, Germany)).

### 4.5. Nanopore Sequencing

gDNA of HepG2 cells was sheared to 10 kb with a Covaris G-Tube and sequenced on MinION flow cells (R9.5) with the 1D Sequencing Genomic Ligation (LSK108) protocol from ONT (Oxford, UK). Briefly, 2µg of sheared gDNA were end repaired and dA-tailed (NEB, MA, USA) and then subsequently cleaned with 1× Ampure XP (Beckman Coulter (Brea, CA, USA)) and eluted in water. End-repaired and dA-tailed DNA was ligated to 1D sequencing adapter with NEBNext Quick Ligation Module using Blunt/TA Ligase Master Mix (NEB). The library was then purified and loaded onto r9.5 flow cells. Reads were base called with Albacore 1.2.6. Nanopore reads were aligned to the hg38 reference genome using Minimap2 [69]. All raw data were submitted to the European Nucleotide Archive (accession PRJEB37910).

### 4.6. Methylation Calling for Nanopore Reads

We determined the methylation status of each CpG site on each read using *nanopolish* call-methylation [17]. Briefly, *nanopolish* uses a five-base alphabet, with 5-methylcytosine represented as M, to build a Gaussian mixture model representing every possible 6-mer with both methylated and unmethylated cytosine in a CpG context, excluding those 6-mers that contain both methylated and unmethylated bases. We ran *nanopolish* separately on our base-called reads. To extract highly confident methylated CpG sites, we set a log-likelihood threshold of 2.5. Coverage and methylation calls were converted into bed and bigwig format for subsequent analysis.

### 4.7. EPIC Sequencing

#### 4.7.1. Library Generation, Quality Control, and Quantification

As input, 500 ng of total DNA per sample were utilized for preparing targeted methylseq libraries with Illumina TruSeq Methyl Capture EPIC Library Preparation Kit (Illumina (San Diego, CA, USA)). All steps were performed as recommended in Illumina user document 1000000001643 v01 May 2017. Furthermore, one additional purification step was introduced at the end of the standard procedure, using 1x Agencourt® AMPure® XP Beads (#A63881; Beckman Coulter, Inc.). The KAPA Hifi HotStart Uracil+Ready Mix 2x enzyme is needed to amplify the enriched libraries but is not substitutable and not included with the kit. DNA libraries were indexed and amplified with 11–13 cycles of PCR. Fragment length distribution of individual libraries was monitored using Bioanalyzer High Sensitivity DNA Assay (5067-4626; Agilent Technologies (Santa Clara, CA, USA). Quantification of libraries was performed by use of the Qubit^®^ dsDNA HS Assay Kit (Q32854; ThermoFisher Scientific). 

#### 4.7.2. Library Denaturation and Sequencing Run

The library pool was denatured with NaOH and was finally diluted to 1.8 pM according to the Denature and Dilute Libraries Guide (Document # 15048776 v02; Illumina). 1.3 mL of the denatured pool was loaded on an Illumina NextSeq 550 sequencer using a High Output Flowcell for paired-end reads (Document 20024907; Illumina). Sequencing was performed with a paired-end sequence with 76 cycles, with a six-base barcode index, and 25% control v3 PhiX. This level of PhiX was required since the samples were relatively GC rich.

#### 4.7.3. Illumina Sequence Data Analysis

Illumina 75bp end-paired datasets were demultiplexed using bcl2fastq version 2.17.1.14 (Illumina). Fastq files were then subjected to quality control with FASTQC and MultiQC and analyzed using the NGI-Methylseq pipeline [70] version 0.4dev with [71]. The genome reference hg38 from Ensembl was used without decoy sequences. 

The pipeline was modified to only make methylation calls for sites covered by at least five reads, instead of the default one read. Coverage and methylation calls were converted into bed and bigwig format for subsequent analysis. Public ENCODE WGBS (non-EPIC) data were processed using the same pipeline. All data were analyzed on the MHH HPC-seq SLURM research cluster. Raw data were submitted to the European Nucleotide Archive (PRJEB37910).

### 4.8. Semi-Quantitative RT-PCR and qRT-PCR Analysis

RNA was isolated from cells with the High Pure RNA Isolation kit (Roche Diagnostics) according to the manufacturer´s instructions. In total, 1µg of RNA was reverse transcribed using oligo dT primer and the SuperScript™ III Reverse Transcriptase (Thermo Fisher Scientific) following the instructions provided. Furthermore, 1/20th of the cDNA mix was used for PCR using 10 pmol of forward and reverse primer pairs for each PCR, which are described in Table S4. The levels of mRNA expression were standardized to the glyceraldehyde-3 phosphate dehydrogenase (GAPDH). 

### 4.9. Glucose-6-Phosphate (Glucose 6-P) Measurement

Overall, 10^6^ cells were homogenized in ice-cold PBS (phosphate-buffered saline) and centrifuged at top speed for 5 min to remove insoluble materials. Cell lysates were aliquoted for protein concentration- and Glucose 6-P measurement. To measure Glucose 6-P, lysates were further deproteinized using 10 kDa molecular weight cutoff spin columns (Abcam, Cambridge, UK). Glucose 6-P measurement was performed with Glucose 6 Phosphate Assay Kit (Colorimetric, Abcam) according to the manufacturer´s instructions. Glucose 6-P was read after 30 minutes at OD 450 nm in a Victor2V 1420 multilabel counter (Perkin Elmer/Wallac, Waltham, MA. USA). Glucose 6-P levels were normalized to protein content (Pierce BCA, 23225).

### 4.10. Lactate Measurement

Lactate levels were measured enzymatically in 96-well plates according to the manufacturer’s specifications (Lactate-Glo Assay, Promega (Madison, WI, USA), J5022). A total of 15000 cells/well were seeded for each measurement. Cell lysates were neutralized with 7.5 μL 1M Tris-base and incubated with a 30 μL detection reagent. Luminescence was recorded after one hour using GloMax® Discover System (Promega), and intracellular lactate concentrations were determined from a standard curve. Lactate levels were normalized to protein content from duplicate plates after lysing cells with Triton X-100 (0.5% *v*/*v*).

### 4.11. NAD+/NADH Measurement

Total NAD (NADt) and NADH levels were measured using a NAD/NADH Quantitation Colorimetric Kit (Biovision (Milpitas, CA, USA)). A total of 5x10^5^ cells were supplied for each measurement according to the manufacturer’s specifications. NADt (NAD+ and NADH) levels were measured were read at OD 450nm in a Victor2V 1420 multilabel counter. NADH was determined by decomposing NAD+ at 60°C, 30 mins before performing detection reaction. NAD+/NADH ratio was determined from the measured NADt and NADH values by the following formula: (NADt-NADH)/NADH.

### 4.12. ATP Measurement

ATP levels were measured in 96-well plates according to the manufacturer’s specifications (ATP Assay Kit (ab83355), Abcam). For this, 10^6^ cells were lysed in ATP lysate buffer and centrifuged at top speed for 5 min to remove insoluble materials. Cell lysates were aliquoted for protein concentration and ATP measurement. To measure ATP, lysates were further deproteinized using 10 kDa molecular weight cutoff spin columns (Abcam, Cambridge, UK). Deproteinized lysates were then supplied to ATP Assay Kit according to the manufacturer’s specifications. ATP level was read at OD 570 nm in a Victor2V 1420 multilabel counter. ATP levels were normalized to protein content.

### 4.13. Crystal Violet Assay

Cells were seeded on a 96-well plate and then washed with PBS, fixed with ice-cold methanol for 10 min on ice, and air dried. After staining with 100 µl of a 0.05% crystal violet solution for 30 min at room temperature, the cells were then washed five times with tap water. Crystal violet was solubilized from the cells in 100 µl methanol, and the absorbance was read at 595 nm in a Victor2V 1420 multilabel counter.

### 4.14. Statistical Analysis

Cell experiments were performed in triplicate, and at least three independent experiments were evaluated. Data were reported as the mean value ± SD. The statistical significance of the difference between groups was determined by the Student’s *t*-test (two-sided). Primary 371 HCC data gathering was limited by availability from the Cancer Genome Atlas (TCGA) data (https://cancergenome.nih.gov/, accessed on 3 February 2021).

## Figures and Tables

**Table 1:** The molecular function of 10 TSG candidates.

Symbol	Description	Potential Function
CXCL12	C-X-C motif chemokine ligand 12	functions as the ligand for the chemokine (C-X-C motif) receptor 4, and plays a role in many diverse cellular functions including cell migration and invasion [36]
EFS	embryonal Fyn-associated substrate	associates with FAK and SRC family kinases to activate downstream effectors regulating the actin cytoskeleton [37]
GCK	glucokinase	phosphorylates glucose and plays a key regulatory role in glucose uptake [38]
KCNQ5	potassium voltage-gated channel subfamily Q member 5	voltage-gated potassium (Kv) channel that regulates changes in membrane potential [39]
NFATC2	nuclear factor of activated T cells 2	functions as transcription factor and involves in NFAT signaling [40]
PLTP	phospholipid transfer protein	involved in transport of lipids and vitamin E among lipoproteins, and between lipoproteins and cells [41]
PRIMA1	proline-rich membrane anchor 1	required for the assembly of Acetylcholinesterase, which plays a crucial role in terminating the synaptic transmission in the central nervous system and at the neuromuscular junctions (NMJs) in the peripheral nervous system [42]
PTH1R	parathyroid hormone 1 receptor	a receptor for parathyroid hormone (PTH) and for parathyroid hormone-like hormone (PTHLH) and may act as proproliferation factor in neuroblastoma [43]
SH3YL1	SH3 and SYLF domain containing 1	coregulator of the Androgen Receptor and participates in Androgen-mediated growth of prostate cancer cells [44]
SYCE1	synaptonemal complex central element protein 1	a member of the synaptonemal complex, which links homologous chromosomes during prophase I of meiosis [45]

## Data Availability

Nanopore and EPIC data are available at the EBI ENA (PRJEB37910). Details of data from all databases and bioinformatic information presented in this study will be shared. Further, all cell lines and materials included here will be available for anyone interested.

## Supplementary Materials

The following are available online at https://www.mdpi.com/article/10.3390/ijms22083937/s1, Table S1: List of 489 potentially hydroxymethylated genes, Table S2: List of 1637 hypermethylated genes, Table S3: List of 56 hypermethylated genes downregulated during liver regeneration, Table S4: Primer table.
