# Supplementary material for: Genome-Wide Methylation Mapping Using Nanopore Sequencing Technology Identifies Novel Tumor Suppressor Genes in Hepatocellular Carcinoma
Source: Int J Mol Sci. 2021 Apr 11;22(8):3937. doi: 10.3390/ijms22083937 (PMC8069345; doi:10.3390/ijms22083937)
Supplement: Supplementary file 1 [file ijms-22-03937-s001.zip › Table S3.docx]

Table S3: 57 hypermethylated-5mC genes downregulated during liver regeneration.

| Gene | Fold downregulation 4h after sham surgery | Fold downregulation 4h after partial hepatectomy (PH) | $\frac{Expression after 1 week PH}{Expression before PH}$ |
| --- | --- | --- | --- |
| Ajap1 | 12.28 | 84.62 | 6.67 |
| Ccdc78 | 44.72 | 368.57 | 48.86 |
| Cd163l1 | 0.94 | 6.14 | 6.14 |
| Celf4 | 1.06 | 15.35 | 1.21 |
| Cldn6 | 3.60 | 36.34 | 1.66 |
| Cxcl12 | 1.83 | 4.19 | 0.85 |
| Ddit4l | 2.22 | 4.99 | 1.00 |
| Dedd2 | 1.07 | 2.79 | 1.22 |
| Dleu7 | 0.91 | 3.92 | 3.92 |
| Dmc1 | 1.00 | 7.12 | 7.12 |
| Efs | 2.56 | 827.82 | 1.99 |
| Gata5 | 1.47 | 5.76 | 2.62 |
| Gck | 1.73 | 3.67 | 1.31 |
| Ggt1 | 4.51 | 10.93 | 9.29 |
| Hcst | 0.25 | 2.82 | 3.70 |
| Hlx | 0.99 | 2.32 | 1.72 |
| Hoxb4 | 0.84 | 2.19 | 1.11 |
| Kcnj2 | 0.72 | 24.21 | 2.27 |
| Kcnq5 | 3.98 | 104.10 | 22.26 |
| Lin37 | 1.23 | 2.75 | 1.25 |
| Lrat | 1.80 | 4.27 | 0.85 |
| Lrch2 | 3.08 | 9.47 | 8.32 |
| Lrrc24 | 0.27 | 3.29 | 0.86 |
| Matk | 0.53 | 3.55 | 1.77 |
| Mcoln3 | 1.81 | 6.19 | 1.05 |
| Mlph | 1.69 | 5.92 | 1.32 |
| Ndufa4l2 | 2.77 | 7.52 | 1.05 |
| Neurl2 | 2.75 | 6.13 | 1.46 |
| Nfatc2 | 1.89 | 11.08 | 1.10 |
| Pbx4 | 1.61 | 5.94 | 1.10 |
| Pde1c | 1.27 | 2.93 | 0.85 |
| Pltp | 1.30 | 3.57 | 1.24 |
| Plxdc1 | 6.25 | 14.72 | 1.10 |
| Prdm16 | 1.26 | 2.92 | 0.91 |
| Prima1 | 1.03 | 2.06 | 2.06 |
| Pth1r | 3.04 | 6.72 | 1.18 |
| Rab33a | 1.77 | 8.87 | 8.87 |
| Sac3d1 | 1.01 | 2.30 | 1.26 |
| Serp2 | 5.25 | 28.94 | 2.44 |
| Sh3yl1 | 1.34 | 3.00 | 1.06 |
| Slc5a11 | 9.02 | 119.20 | 13.48 |
| Sorbs3 | 1.37 | 2.77 | 1.21 |
| Sox30 | 16.34 | 53.86 | 8.42 |
| Spo11 | 1.67 | 9.46 | 9.46 |
| St8sia2 | 2.73 | 29.80 | 4.04 |
| Syce1 | 1.34 | 9.68 | 1.23 |
| Sycp3 | 2.04 | 38.74 | 38.74 |
| Tbx6 | 2.87 | 6.26 | 1.48 |
| Tcf15 | 2.01 | 19.26 | 19.26 |
| Tmem215 | 2.37 | 34.42 | 38.08 |
| Tmem91 | 6.66 | 38.13 | 6.52 |
| Trim36 | 0.53 | 4.63 | 4.71 |
| Tspan10 | 0.31 | 7.31 | 1.06 |
| Twist1 | 0.60 | 183.79 | 0.98 |
| Vash2 | 1.51 | 16.97 | 2.76 |
| Zfyve28 | 3.78 | 7.73 | 7.56 |
