# Supplementary material for: Genome-Wide Methylation Mapping Using Nanopore Sequencing Technology Identifies Novel Tumor Suppressor Genes in Hepatocellular Carcinoma
Source: Int J Mol Sci. 2021 Apr 11;22(8):3937. doi: 10.3390/ijms22083937 (PMC8069345; doi:10.3390/ijms22083937)
Supplement: Supplementary file 1 [file ijms-22-03937-s001.zip › Table S4.doc]

**Table S4:** PCR primer pair sequences for selected genes.

| **Gene** | **Accession number** | **Forward primer** | **Reverse primer** | **PCR product (bp)** |
| --- | --- | --- | --- | --- |
| ***GAPDH*** | NM_002046 | TGTTGCCATCAATGACCCCTT | CTCCACGACGTACTCAGCG | 202 |
| ***GCK*** | NM_0001625 | CCTCACTTCCCTGGGAACTCAT | TCAGGCAAGAAAATGGCCAGGACT | 126 |
| ***MCT1/*** ***SLC16A1*** | NM_003051.4 | GGTGGAGGTCCTATCAGCAGT | CAGAAAGAAGCTGCAATCAAGC | 107 |
| ***MCT2/*** ***SLC16A7*** | NM_001270623.2 | GCAACCCGCCTTAACCATAAT | AGGTACTGATTGAAAGGAGCCA | 120 |
| ***MCT4/*** ***SLC16A3*** | NM_004207.4 | CGGCTTTGTGCTTTACGCC | GCTGAAGAGGTAGACGGAGTA | 220 |
